# No Association of the BDNF Val66met Polymorphism with Implicit Associative Vocabulary and Motor Learning

**DOI:** 10.1371/journal.pone.0048327

**Published:** 2012-11-13

**Authors:** Nils Freundlieb, Stephan Philipp, Susanne A. Schneider, Norbert Brüggemann, Christine Klein, Christian Gerloff, Friedhelm C. Hummel

**Affiliations:** 1 Brain Imaging and Neurostimulation (BINS) Laboratory, Department of Neurology, University Medical Center Hamburg-Eppendorf, Hamburg, Germany; 2 Schilling Section of Clinical and Molecular Neurogenetics, Department of Neurology, University of Luebeck, Luebeck, Germany; Julius-Maximilians-Universität Würzburg, Germany

## Abstract

Brain-derived neurotrophic factor (BDNF) has been suggested to play a major role in plasticity, neurogenesis and learning in the adult brain. The *BDNF* gene contains a common val66met polymorphism associated with decreased activity-dependent excretion of BDNF and a potential influence on behaviour, more specifically, on motor learning. The objective of this study was to determine the influence of the *BDNF* val66met polymorphism on short-term implicit associative learning and whether its influence is cognitive domain-specific (motor vs. language). A sample of 38 young healthy participants was genotyped, screened for background and neuropsychological differences, and tested with two associative implicit learning paradigms in two different cognitive domains, i.e., motor and vocabulary learning. Subjects performed the serial reaction time task (SRTT) to determine implicit motor learning and a recently established associative vocabulary learning task (AVL) for implicit learning of action and object words. To determine the influence of the *BDNF* polymorphism on domain-specific implicit learning, behavioural improvements in the two tasks were compared between val/val (n = 22) and met carriers (val/met: n = 15 and met/met: n = 1). There was no evidence for an impact of the *BDNF* val66met polymorphism on the behavioural outcome in implicit short-term learning paradigms in young healthy subjects. Whether this polymorphism plays a relevant role in long-term training paradigms or in subjects with impaired neuronal plasticity or reduced learning capacity, such as aged individuals, demented patients or patients with brain lesions, has to be determined in future studies.

## Introduction

Brain-derived neurotrophic factor (BDNF) is one of the most abundant neurotrophic factors in the adult brain, associated with development, synaptic plasticity and learning [1]–[3]. It is highly expressed in CNS structures including the cortex, the hippocampus and limbic structures [4], [5]. A single nucleotide polymorphism (SNP) at codon 66 leads to an exchange of valin to methionin (val66met Polymorphism) resulting in three genotypes (homocygote val/val or met/met and heterocygote val/met) with varying distribution worldwide [6]. Due to low numbers of met/met, met-carriers (met/met and val/met) are mostly analysed together [2], [7], [8]. In German cohorts, a ratio of approximately 60% (val/val) to 40% (met-carrier) was found in earlier studies [9], [10].

While the polymorphism does not alter the mature BDNF structure, it impairs its trafficking resulting in: 1) decreased variant BDNF distribution into neuronal dendrites; 2) decreased targeting to secretory granules and 3) subsequent impairment of regulated, activity-dependent secretion [2], [5]. These defects have been associated with neuroanatomical and behavioural differences between young healthy subjects, e.g., hippocampal volume [11] and function (i.e. episodic memory [2], [12]) is decreased in met-carriers. Studies assessing other memory domains (i.e. working memory [13], especially tests which rely less on hippocampal function or integrity, such as planning tasks [2], [3] have shown differing results.

Due to the expression of BDNF in several brain structures including the cortex, it is tempting to hypothesize that the polymorphism might affect various memory or learning systems. For example, in bipolar disorder patients, met-carriers performed worse on the Wisconsin card sorting task, a task that tests disorders of executive frontal lobe functions [14].

Controversy still exists about the role of the polymorphism for motor learning: Using fMRI and non-invasive brain stimulation, it has been suggested that the polymorphism is associated with short-term plasticity of the motor cortex [15]–[19]. Nonetheless, other studies with only slightly differing protocols failed to replicate this association [20], [21]. Regarding differential effects on motor behaviour and more specifically motor learning, some studies were able to show effects of genotype on short-term or long-term learning [8], [15], whereas other studies failed to show such an influence of the polymorphism [16], [18], [20].

Therefore, we aimed to determine whether the *BDNF* polymorphism affects cortical learning systems and whether such an effect is domain-specific or not while controlling for potential confounders such as age, ethnicity and mood changes. To this aim, two learning domains with high relevance on daily life were chosen, that are motor and language learning. Thirty-eight young healthy students were genotyped, characterized with an extensive neuropsychological screening and tested with two behavioural tasks in different cognitive domains: (a) the serial reaction time task (SRTT) as a measure for implicit motor learning and (b) an associative implicit vocabulary learning task (AVL) for the language domain.

## Materials and Methods

All subjects gave written informed consent to participate in the experiment according to the Code of Ethics of the World Medical Association before all experimental procedures. The study was approved by the local ethics committee (Ethik-Kommission der Ärztekammer Hamburg, Germany).

### Subjects

Thirty-eight young healthy right-handed subjects (26 females, mean age: 24.0±0.3 years; age range: 22–27years) participated in the study. Previous studies addressing the effects of BDNF on either motor learning or non-invasive brain stimulation were based on comparatively small group sizes (total group size: median = 24 persons (range 12–36); met-carrier group size: median 7.5 (range 7–18) [8], [15]–[22]). Thus, we chose a group size of 38 subjects, twice as much as the average, to be able to monitor an Effect size d∼0.5 with a power of 0.3. According to the Edinburgh Inventory of Handedness [23], all subjects were right-handed and were naïve to the experimental purpose of the study. None of the subjects had a history of serious medical, neurological or psychiatric illness, or used illegal, neuroactive or recreational drugs (>15 cigarettes/day, >6 cups of coffee/day, >50 g of alcohol/day) as probed by a standardized questionnaire. All were of Caucasian ethnicity, students of medicine at the University of Hamburg, Germany with their first and only native language German and spoke 1–4 foreign languages (mean: 2.24±0.85).

### Experimental Protocol

Study participation consisted of two sessions: During the first session subjects underwent standardized neuropsychological tests to assure homogeneity concerning visuospatial memory and executive abilities (Rey-Osterrieth Complex Figure Test [24], attention (d2 test [25]), working memory (digit spans), verbal learning ability (VLMT: verbal learning and memory test [26]), verbal fluency (Regensburg verbal fluency test: formal and semantic subtest [27] and logical reasoning (Horn intelligence test, subtest 4 [28]). We used the Beck Depression Inventory (BDI [29]) to screen for and exclude depression. Additionally, average weekly hours playing a musical instrument or using the computer were recorded. Then, a blood sample for genotyping was obtained.

During the second session, subjects underwent two tests evaluating their implicit learning abilities within the motor (SRTT) and the language domain (associative vocabulary learning (AVL)) in a double-blinded manner. Before SRTT, between both tests and after AVL subjects were controlled for their subjective positive and negative feelings, using the Positive and Negative Affective scale (PANAS). The PANAS consists of two 10-item mood scales that assess the dimensions of positive and negative affect. Both sessions were separated by at least one night. Subjects who scored above 14 in the BDI or more than 2 SD below or above the mean in two of the neuropsychological tests were excluded from the study.

### Serial Reaction Time Task (SRTT)

The SRTT, originally developed by Nissen and Bullemer [30], was slightly modified to test implicit sequence learning. The task is a choice reaction time task with four possible responses carried out with four different fingers. Subjects were seated in a comfortable office chair in front of a 17inch flat screen display at eye level and a standard computer keyboard with German layout. They were instructed in a standardized manner to press the keys “v” (index finger), “g” (middle finger), “h” (ring finger), “m” (small finger) as fast and as correct as possible corresponding to the appearance of an asterisk in one of the 4 positions that were horizontally spaced on the screen. Each asterisk was presented for 1000 ms, which was also the time frame for key presses, intertrial interval consisted of 500 ms. The length of pause between blocks was determined by the participant. Reaction time for each key press was recorded, too early and too late answers (faster than 150 ms or slower than 850 ms) were counted as misses.

A session consisted of 8 blocks with 120 trials each. In blocks 1 and 6 the sequence of asterisks followed a pseudo-random order whereas in the other blocks, the same 12-trial sequence of asterisk position was repeated 10 times. Subjects were not told about the repeating sequence, but asked after the last block whether they could recall a repeating sequence. Whereas a decrease of reaction time is considered as improvement of general visuomotor performance, difference between sequential and random block response times (ΔRT_block5_/RT_block6_) and (ΔRT_block6_/RT_block7_) are regarded as a measure for implicit motor learning [31].

### Associative Vocabulary Learning (AVL)

AVL was tested as previously described [32], [37]. In brief, pictures of concrete, body-related actions (e.g. eat) and static objects (e.g. house) were combined with meaningless pseudowords. Seventeen objects and 17 actions, each represented by two different photos, were randomly assigned to one of 34 pseudowords (“correct” coupling). During learning, the correct coupling was presented ten times, whereas each object and action was also presented once with a total of ten different pseudowords (“incorrect” coupling, correct-incorrect ratio 10:1). This resulted in a total of 680 trials which were divided into 5 blocks of 136 trials each. The order of trials was pseudorandomized, so that the same action, object, pseudoword, stimulus class (object or action) or type of coupling (correct or incorrect) would appear maximally three times consecutively.

For testing, subjects were instructed in a standardized manner to decide intuitively whether action or object and pseudoword matched (pressing the left mouse button with the right index finger) or not (pressing the right mouse button with the right middle finger). They were also told that they had to respond before the picture disappeared (1400 ms). Responses after time-out were scored as error. No feedback about their success rate was provided during learning.

During training, photos of 5.4 cm^2^ were presented at eye level 200 ms after onset of acoustically presented pseudowords. Reaction times were recorded from onset of the picture until first response or disappearance of the picture (1400 ms) for all trials separately. The inter-trial interval was 2 s. After the training session, all 34 pseudowords were presented twice (with a 2 s inter-trial interval) without pictures. Participants were asked to translate the pseudowords into German.

### BDNF- Genotyping

Genotyping of BDNF val66met was performed by a DNA melting curve analysis with variant-specific probes on the LightCycler® (RocheDiagnostics; Mannheim, Germany). Primer sequences were forward: 5′-CCAGGTGAGAAGAGTGATGAC-3′ and reverse: 5′-GGCACTTGACTACTGAGCATC-3′.

Following hybridization probes were used: 5′-LC LC640-CGAACACATGATAGAAGAGCTGTT-3′-phosphate (anchor probe) and 5′-AAGAGGCTTGACATCATTGGCTGACACT-3′fluorescein (sensor probe). Participants were divided into two groups according to their genotype, either (i) homozygous for the val allele (val/val) or (ii) homozygous and heterozygous for the met allele (met/met, val/met), respectively. The examiners and subjects were blinded with respect to the genotype.

## Data Analysis

### Subjects

Demographics and data of the neuropsychological tests were examined between the two genotype groups using two-tailed t-tests.

### SRTT

In each trial, response time (RT) was recorded from the appearance of the asterisk until the first button was pushed by the subject. For each block of trials, mean RT was calculated for each subject separately. Incorrect responses and RTs of less than 150 ms or more than 850 ms were discarded. A two-way repeated measures analysis of variance (rmANOVA) with the factor “time” (8 levels: Block 1–8) and “genotype” (2 levels: val/val vs. met-carrier) was used to determine differences in implicit motor learning. Since RT differences between random and sequence blocks are thought to represent an exclusive measure of implicit learning, differences of RT_block5_ and RT_block6_ and between RT_block6_ andRT_block7_ were tested using a two-sided student’s t-test. To test for differences between the genotypes, two-sided Student’s t-tests were then performed to compare the differences of (ΔRT_block5_/RT_block6_) and (ΔRT_block6_/RT_block7_) between the genotype groups.

### AVL

To address successful learning in the AVL paradigm, the percentage of correct answers in the translation test was compared using a two-sided t-test. We additionally analysed learning success over time (i.e. percentage of correct decisions) and RT using a two-way rmANOVA involving the factors “time” (5 levels: Block 1–5) and “genotype” (2 levels: val/val vs. met-carrier). Furthermore, responses were analysed separately for action words and object words.

As control parameters, we analyzed the scores of the neuropsychological tests, PANAS as well as the RT of the simple motor task.

Kolmogorov-Smirnov tests for normal distribution were calculated before statistical parametric testing was applied.

All ANOVA results were Greenhouse-Geisser corrected when sphericity was violated. Bonferroni-corrected t-tests were used for post-hoc tests. Results were considered significant at a level of p<0.05. All data are expressed as mean ± standard error. Statistical analyses were done using SPSS 17.0®.

## Results

Neither demographic nor neuropsychological measures differed between the two genotype groups (Table 1). No subject had to be excluded due to a high score in BDI. PANAS did not detect any changes in positive scores (time: F_2_ = 1.127; p = 0.31; time*genotype: F_2,72_ = 0.081; p = 0.84) nor in negative scores (time: F_2_ = 0.16; p = 0.835; time*genotype: F_2,72_ = 0.56; p = 0.56).

**Table 1:** Subjects demographics and neuropsychological scores.

	val/val (n = 22)	met-carriers (n = 16)
sex (f/m)	12/10	14/2
age (yrs)	24.1±0.3	23.9±0.5
Number of spoken foreign languages	2.3±0.2	2.1±0.2
Musical instrument hrs/wk	1.0±0.5	0.3±0.2
Keyboard writing hrs/wk	3.9±1.0	6.3±2.2
Oldfield Handedness Score	0.9±0.0	0.8±0.0
Language score	5.6±0.4	5.1±0.6
d2 KL (concentration)	200.6±7.1	200.7±8.4
Beck’s Depression Inventory	2.7±0.5	3.9±1.2
VLMT DG1-5 RW	63.4±0.9	63.6±1.4
VLMT DG7 RW	14.2±0.3	14.0±0.4
VLMT DG5-DG7 RW	0.2±0.3	0.4±0.4
VLMT W-F RW	14.6±0.1	14.6±0.3
Digit span	14.4±0.5	14.2±0.8
Logical reasoning	27.9±0.9	27.4±0.8
Rey complex figure copy	35.8±0.1	35.8±0.1
Rey complex figure immediate reproduction	25.3±1.4	28.1±0.8
Rey complex figure 30 min	25.1±1.4	27.7±0.9
Verbal fluency “animal”	41.8±1.9	39.7±1.9
Verbal fluency letter “s”	29.6±1.1	27.6±2.2
Verbal fluency sum	71.4±2.7	67.3±3.3

all p>0.05 except for gender (p = 0.023).

### SRTT

**Figure 1:**
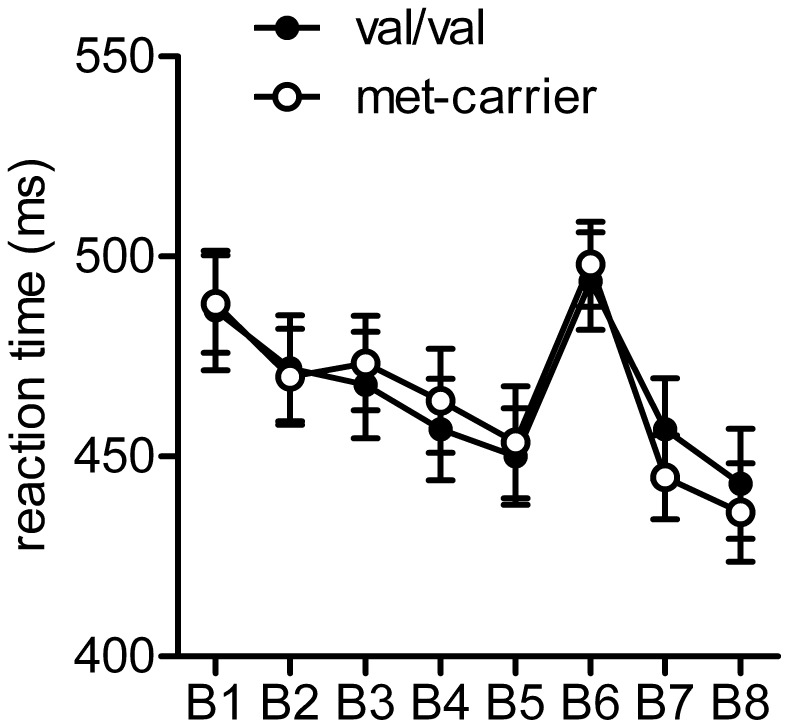
No significant difference between the genotypes regarding the reaction times (ms) during SRTT.

While factor “time” was significant F_7_ = 4.65, p<0.001; the “time” * ”genotype” interaction was not (F_7,252_ = 0.68; p = 0.63, see Fig 1). Post-hoc t-tests showed significant differences of RT between the random block 6 and its surrounding sequential blocks (block 5 and block 7) (Reaction time (RT)_block6_ = 495.7 ms±8.2 vs. RT_block5_ = 451.6 ms±9.0 (T = −9.78; p<0.001) and RT_block7_ = 451.8 ms±8.5 (T = 8.80; p<0.001).

No significant differences were found between genotypes regarding ΔRT_block5_/RT_block6_ (val/val: 43.4 ms±4.2; met-carrier: 44.4 ms±9.6; T = −0.10; p = 0.92) and ΔRT_block6_/RT_block7_ (val/val:−38.0 ms±7.0; met-carrier: 54.2 ms±6.2; t-test: p = 0.09). After testing, some of the participants were conscious about a repetitive sequence, an effect similar within both genotype groups, although none of them could recall the whole sequence.

### AVL

**Figure 2:**
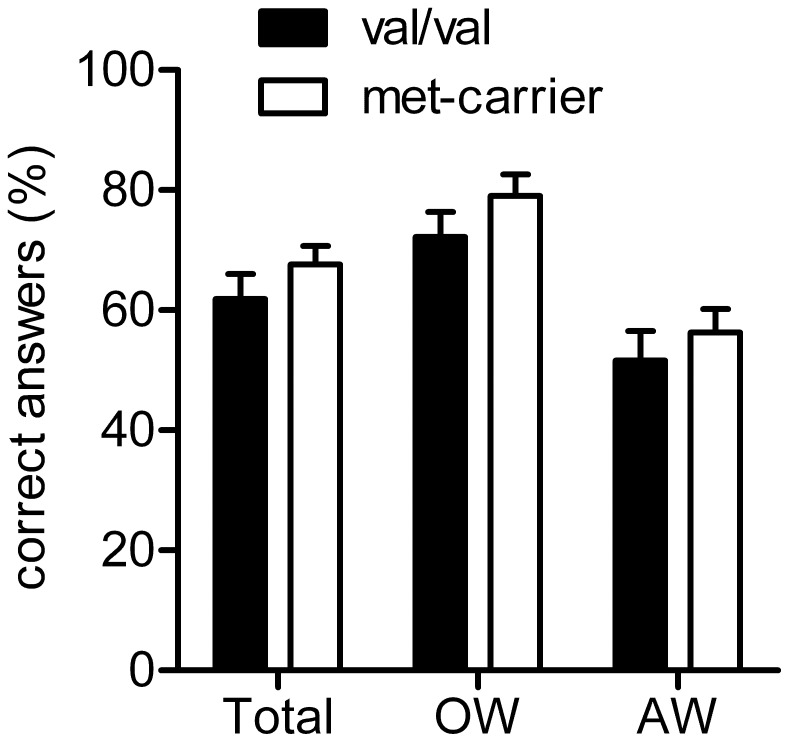
Translation rate after training does not differ between BDNF genotypes (total and for Object words (OW) and Action words (AW)).

Translation rate did not differ between the two genotype groups, neither totally nor separated for action words and object words (Total: val/val: 61.90%±4.18; met-carrier: 67.65%±3.05; Object words only: val/val: 72.19%±4.21; met-carrier: 79.05%±3.60; Action words only: val/val: 51.60%±4.93; met-carrier: 56.25%±3.98; all p>0.1; Fig 2). Subjects started at 44.9%±1.0 correct answers in Block 1 (val/val: 44.1%±1.4; met-carrier: 46.1%±1.4; p = 0.32) and reached a total of 84.2%±1.5 (val/val: 83.1%±2.3; met-carrier: 85.7%±1.6; p = 0.38).

**Figure 3:**
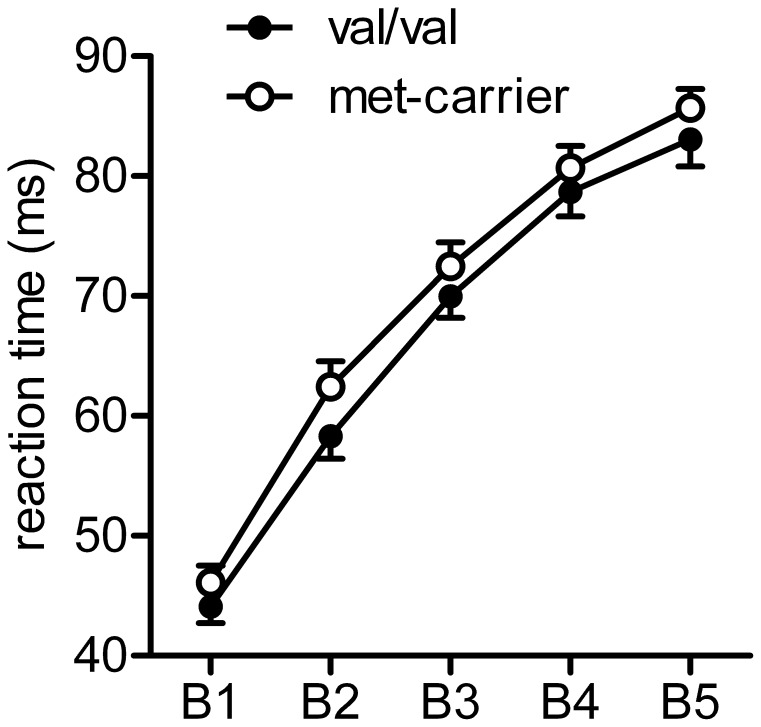
Both genotype groups show similar learning success during associative vocabulary learning.

Regarding learning success, an rmANOVA revealed that “time” was significant (F_4_ = 70.21; p<0.001), but there were no significant differences between the two genotype groups (F_4,140_ = 0.75; p = 0.6) (Fig 3). Similarly for RT, factor “time” was significant (F_4_ = 41.30; p = <0.001) whereas “time” * “genotype” was not (F_4,140_ = 2.24; p = 0.11).

## Discussion

Brain derived neurotrophic factor (BDNF) is widely expressed in the adult hippocampus and neocortex [4]. It is considered one of the most abundant neurotrophic factors and important for neuronal differentiation and life-long plasticity and repair [5]. A single nucleotide polymorphism exchanging valin to methionin (*BDNF* val66met polymorphism) impairs activity-dependent BDNF excretion and has been associated with changes in cortical and subcortical anatomy [2], [11], [15]. Thus, it was hypothesized that the genotype might also influence the behavioural phenotype.

The aim of the present study was therefore to evaluate the influence of the BNDF val66met polymorphism on implicit short-term learning and whether its effect is dependent of the cognitive domain tested. As representative cognitive domains, we chose to test the motor and the language domain which are both highly relevant in everyday life. The motor domain was explored by using the SRTT, a well-established task for implicit motor learning [19], [31]. Secondly, implicit language learning was addressed using a recently developed associative action and object word learning paradigm [32].

To exclude as many potential confounders as possible, participants were tested in a double-blinded manner; stratified for age, educational background, ethnicity and were also monitored during testing for mood changes. They were also characterized in an extensive neuropsychological evaluation to rule out significant background differences regarding verbal learning ability, verbal fluency, visuospatial abilities, attention span, working memory or pre-existent depressive traits. With these strict inclusion and exclusion criteria the study group was well homogenous. Within this neuropsychologically well characterized homogenous group no behavioural or learning differences during the tested tasks could be determined for the *BDNF* polymorphism. The subjects showed clear implicit learning during the SRTT and the AVL, however implicit learning was not influenced by the *BDNF* genotype (val/val vs. met-carrier) as suggested by a recent study [33]. During the SRTT reaction times, considered as markers of performance, decreased in a comparable manner, also all measures for implicit learning, i.e., the differences between the sequential and random blocks, were not significantly influenced by *BDNF*. Similarly, within the AVL both groups started at similar levels and reached a comparable magnitude of improvement during learning without any difference due to the *BDNF* polymorphism.

In contrast to previous work [8], [15], we did not see any effects of the *BDNF* polymorphism on motor learning. There are several reasons which might potentially explain this: (a) the present number of subjects is in the range of recent other motor learning studies [15], [16], [20], nevertheless compared to studies evaluating the effect of the *BDNF* genotype in other domains (e.g. 785 subjects [13]; 641 subjects [2]) the sample size is rather small. (b) A single training session approach was used, thus short-term motor learning was evaluated. The few studies of *BDNF* and short-term motor learning showed heterogeneous results. McHughen et al. (2010) revealed in a 15 min driving-based motor learning task that met-carrier showed less short-term learning with an enhanced error rate and lower retention after 4 days [15]. Another recent study by Joundi et al. (2012) evaluated adaptation learning and described reduced levels of adaptation learning especially at the follow-up session after 24 hours suggested to reflect impaired ‘savings’ mechanisms within the met-carriers [34]. However other studies did not reveal any difference between the genotypes for different motor learning tasks [16], [18], [20]. The present results are in line with these studies. Excretion of *BDNF* and consequent dendritic or synaptic changes need at least several minutes of stimulation [35], which might explain why short-term motor learning tests or non-invasive brain stimulation techniques like iTBS might fail to detect any differences between the genotypes. In contrast, long-term training with repetitive sessions revealed differences between both polymorphisms as demonstrated in recent work [8]. During an isometric sequential pinch force task trained over five consecutive days, differences between Val/Val- and Met-carriers appeared approximately after two days of training and persisted throughout the training sessions [8]. Interestingly, this data could be replicated in a knock-out mouse model [8]. (c) Our study and most of the previously published studies focussed on young healthy individuals. Little is known however about the influence of the *BDNF* polymorphism on the ageing brain. Working memory might be reduced in healthy older met-carriers [2], [36] but not in adolescent met-carriers [13] pointing to age-dependent changes in the rate of influence. This is in line with findings in other polymorphisms [37] and led to the hypothesis that ageing magnifies genetic effects due to decreased compensatory mechanisms [38]. (d) As in the present study males and females were not completely matched in the two *BDNF* groups, an effect of gender cannot be completely ruled out. However in comparable experimental paradigms (AVL) we didn’t see any gender effects on performance [32], [39].

The present data is to our knowledge the first testing the influence of the *BDNF* polymorphism on a non-motor associative learning task, the AVL. Also in this non-motor, language task, we did not see any differences between Val/Val- and Met-carriers. There were no cognitive domain specific differences of the effects of the *BDNF* polymorphism detectable. Therefore, the explanations for a lack of a *BDNF* specific effect on the AVL might be similar like the ones discussed above for the SRTT.

Although data on the influence of the *BDNF* val66met polymorphism on behavioural measures in humans are heterogeneous, there is clear evidence that electrophysiological parameters as well as the efficacy of interventions modulating cortical excitability and neuroplasticity are influenced by this polymorphism. Kleim et al. (2006) showed in a landmark study that met-carriers have a smaller increase of motor cortical reorganisation after a simple motor task [16]. The after-effects of LTP/LTD-like protocols induced by iTBS or cTBS were reported to be reduced or even absent in met-carriers [17]. Accordingly, a study analysing data retrospectively for genotype differences showed similar results [19]. Furthermore, pharyngeal electric stimulation increased MEP amplitude more in val/val than in met-carrier [22].

In subjects with an impaired sensorimotor system, due to age or focal brain lesions, with reduced compensatory mechanisms and/or the necessity to extensive neuroplastic changes, the relevance of the *BDNF* polymorphism might be much bigger, especially in the view that “(re-)learning” is one of the basic principles for successful recovery from neurological diseases such as stroke. The *BDNF* val66met polymorphism has been associated with poor outcome after intracerebral bleeding [40] and it has been speculated that it might influence recovery after stroke [41], [42]. An altered susceptibility has been shown in other neuropsychiatric diseases, such as Alzheimer’s disease [43], depression [44], eating disorders [45], and bipolar disorder [46].

Apart from large genetic studies including hundreds of patients, it seems intriguing to test neuronal plasticity, learning and the potential of recovery in well defined samples of aged people, patients suffering from stroke or other neurologic diseases to provide the basis for future genotype-based personalized treatment strategies.

## References

[1] Poo MM (2001) Neurotrophins as synaptic modulators. Nat Rev. Neurosci 2: 24–32. 10.1038/35049004 11253356

[2] Egan MF, Kojima M, Callicott JH, Goldberg TE, Kolachana BS, et al (2003) The BDNF val66met polymorphism affects activity-dependent secretion of BDNF and human memory and hippocampal function. Cell 112: 257–269. 12553913 10.1016/s0092-8674(03)00035-7

[3] Hariri AR, Goldberg TE, Mattay VS, Kolachana BS, Callicott JH, et al (2003) Brain-derived neurotrophic factor val66met polymorphism affects human memory-related hippocampal activity and predicts memory performance. J Neurosci 23: 6690–6694. 12890761 10.1523/JNEUROSCI.23-17-06690.2003 PMC6740735

[4] Huang EJ, Reichardt LF (2001) Neurotrophins: roles in neuronal development and function. Ann Rev Neurosci 24: 677–736. 11520916 10.1146/annurev.neuro.24.1.677 PMC2758233

[5] Bath KG, Lee FS (2006) Variant BDNF (Val66Met) impact on brain structure and function. Cogn Affect Behav Neurosci 6: 79–85. 16869232 10.3758/cabn.6.1.79

[6] Shimizu E, Hashimoto K, Iyo M (2004) Ethnic difference of the BDNF 196G/A (val66met) polymorphism frequencies: the possibility to explain ethnic mental traits. Am J Med Genet B Neuropsychiatr Genet 126B: 122–123. 15048661 10.1002/ajmg.b.20118

[7] Soliman F, Glatt CE, Bath KG, Levita L, Jones RM, et al (2010) A genetic variant BDNF polymorphism alters extinction learning in both mouse and human. Science 327: 863–866. 20075215 10.1126/science.1181886 PMC2829261

[8] Fritsch B, Reis J, Martinowich K, Schambra HM, Ji Y, et al (2010) Direct Current Stimulation Promotes BDNF-Dependent Synaptic Plasticity: Potential Implications for Motor Learning. Neuron 66: 198–204. 20434997 10.1016/j.neuron.2010.03.035 PMC2864780

[9] Schüle C, Zill P, Baghai TC, Eser D, Zwanzger P, et al (2006) Brain-derived neurotrophic factor Val66Met polymorphism and dexamethasone/CRH test results in depressed patients. Psychoneuroendocrinology 31: 1019–1025. 16890377 10.1016/j.psyneuen.2006.06.002

[10] Gajewski PD, Hengstler JG, Golka K, Falkenstein M, Beste C (2011) The Met-allele of the BDNF Val66Met polymorphism enhances task switching in elderly. Neurobiol Aging 32: 2327 e7–19. 10.1016/j.neurobiolaging.2011.06.010 21803453

[11] Pezawas L, Verchinski BA, Mattay VS, Callicott JH, Kolachana BS, et al (2004) The brain-derived neurotrophic factor val66met polymorphism and variation in human cortical morphology. J Neurosci 24: 10099–10102. 15537879 10.1523/JNEUROSCI.2680-04.2004 PMC6730170

[12] Ho B-C, Milev P, O’Leary DS, Librant A, Andreasen NC, et al (2006) Cognitive and magnetic resonance imaging brain morphometric correlates of brain-derived neurotrophic factor Val66Met gene polymorphism in patients with schizophrenia and healthy volunteers. Arch Gen Psychiatry 63: 731–740. 16818862 10.1001/archpsyc.63.7.731 PMC3065118

[13] Hansell NK, James MR, Duffy DL, Birley AJ, Luciano M, et al (2007) Effect of the BDNF V166M polymorphism on working memory in healthy adolescents. Genes Brain Behav 6: 260–268. 16848784 10.1111/j.1601-183X.2006.00254.x

[14] Rybakowski JK, Borkowska A, Skibinska M, Hauser J (2006) Illness-specific association of val66met BDNF polymorphism with performance on Wisconsin Card Sorting Test in bipolar mood disorder. Mol Psychiatry 11: 122–124. 16222333 10.1038/sj.mp.4001765

[15] McHughen SA, Rodriguez PF, Kleim JA, Kleim ED, Marchal Crespo L, et al (2010) BDNF val66met polymorphism influences motor system function in the human brain. Cereb Cortex 20: 1254–1262. 19745020 10.1093/cercor/bhp189 PMC2852510

[16] Kleim JA, Chan S, Pringle E, Schallert K, Procaccio V, et al (2006) BDNF val66met polymorphism is associated with modified experience-dependent plasticity in human motor cortex. Nat Neurosci 9: 735–737. 16680163 10.1038/nn1699

[17] Cheeran B, Talelli P, Mori F, Koch G, Suppa A, et al (2008) A common polymorphism in the brain-derived neurotrophic factor gene (BDNF) modulates human cortical plasticity and the response to rTMS. J Physiol 586: 5717–5725. 18845611 10.1113/jphysiol.2008.159905 PMC2655403

[18] McHughen SA, Pearson-Fuhrhop K, Ngo VK, Cramer SC (2011) Intense training overcomes effects of the val(66)met BDNF polymorphism on short-term plasticity. Exp Brain Res 213: 415–422. 21769545 10.1007/s00221-011-2791-z

[19] Antal A, Chaieb L, Moliadze V, Monte-Silva K, Poreisz C, et al (2010) Brain-derived neurotrophic factor (BDNF) gene polymorphisms shape cortical plasticity in humans. Brain Stimul 3: 230–237. 20965453 10.1016/j.brs.2009.12.003

[20] Li Voti P, Conte A, Suppa A, Iezzi E, Bologna M, et al (2011) Correlation between cortical plasticity, motor learning and BDNF genotype in healthy subjects. Exp Brain Res 12: 91–99. 10.1007/s00221-011-2700-5 21537966

[21] Nakamura K, Enomoto H, Hanajima R, Hamada M, Shimizu E, et al (2011) Quadri-pulse stimulation (QPS) induced LTP/LTD was not affected by Val66Met polymorphism in the brain-derived neurotrophic factor (BDNF) gene. Neurosci Lett 487: 264–267. 20970479 10.1016/j.neulet.2010.10.034

[22] Jayasekeran V, Pendleton N, Holland G, Payton A, Jefferson S, et al (2011) Val66Met in Brain-Derived Neurotrophic Factor Affects Stimulus-Induced Plasticity in the Human Pharyngeal Motor Cortex. Gastroenterology 141: 827–836.e1–3. 21699787 10.1053/j.gastro.2011.05.047

[23] Oldfield RC (1971) The assessment and analysis of handedness: the Edinburgh inventory. Neuropsychologia 9: 97–113. 5146491 10.1016/0028-3932(71)90067-4

[24] Rey A (1959) Manuel du test de copie d’une figure complexe de A. Rey. Paris: Les Editio.

[25] Brickenkamp R (2002) Test d2 - Aufmerksamkeits-Belastungs-Test. Göttingen: Hogrefe.

[26] Helmstaedter C, Lendt M, Lux S (2001) Verbaler Lern-und Merkfähigkeitstest. Göttingen: Beltz Test.

[27] Aschenbrenner S, Tucha O, Lange K (2000) Wortflüssigkeits-Test. Göttingen: Hogrefe.

[28] Horn W (1983) Leistungsprüfsystem. Göttingen: Hogrefe.

[29] Beck AT (1995) Beck-Depressions-Inventar. Bern: Verlag Hans Huber.

[30] Nissen MJ, Bullemer P (1987) Attentional requirements of learning: evidence from performance measures. Cogn Psychol 19: 1–32.

[31] Robertson EM (2007) The serial reaction time task: implicit motor skill learning? J Neurosci 27: 10073–10075. 17881512 10.1523/JNEUROSCI.2747-07.2007 PMC6672677

[32] Liuzzi G, Freundlieb N, Ridder V, Hoppe J, Heise K, et al (2010) The involvement of the left motor cortex in learning of a novel action word lexicon. Curr Biol 20: 1745–1751. 20888226 10.1016/j.cub.2010.08.034

[33] Witte A V, Kurten J, Jansen S, Schirmacher A, Brand E, et al (2012) Interaction of BDNF and COMT polymorphisms on paired-associative stimulation-induced cortical plasticity. J Neurosci 32: 4553–61. 22457502 10.1523/JNEUROSCI.6010-11.2012 PMC6622078

[34] Joundi RA, Lopez-Alonso V, Lago A, Brittain JS, Fernandez-Del-Olmo M, et al.. (2012) The effect of BDNF val66met polymorphism on visuomotor adaptation. Exp Brain Res. Sep 1. [Epub ahead of print]. 10.1007/s00221-012-3239-9 22941316

[35] Tanaka J-I, Horiike Y, Matsuzaki M, Miyazaki T, Ellis-Davies GC, et al (2008) Protein synthesis and neurotrophin-dependent structural plasticity of single dendritic spines. Science 319: 1683–1687. 18309046 10.1126/science.1152864 PMC4218863

[36] Dempster E, Toulopoulou T, McDonald C, Bramon E, Walshe M, et al (2005) Association between BDNF val66 met genotype and episodic memory. Am J Med Genet B, Neuropsychiatr Genet 134B: 73–75. 15719396 10.1002/ajmg.b.30150

[37] Lindenberger U, Nagel IE, Chicherio C, Li SC, Heekeren HR, et al (2008) Age-related decline in brain resources modulates genetic effects on cognitive functioning. Front Neurosci 2: 234–244. 19225597 10.3389/neuro.01.039.2008 PMC2622748

[38] Nagel IE, Chicherio C, Li S-C, von Oertzen T, Sander T, et al (2008) Human aging magnifies genetic effects on executive functioning and working memory. Front Hum Neurosci 2: 1. 18958202 10.3389/neuro.09.001.2008 PMC2525971

[39] Freundlieb N, Ridder V, Dobel C, Enriquez-Geppert S, Baumgaertner A, et al (2012) Associative vocabulary learning: Development and testing of two paradigms for the (re-) acquisition of action- and object-related words. Plos ONE 7: e37033. 22701562 10.1371/journal.pone.0037033 PMC3368912

[40] Siironen J, Juvela S, Kanarek K, Vilkki J, Hernesniemi J, et al (2007) The Met allele of the BDNF Val66Met polymorphism predicts poor outcome among survivors of aneurysmal subarachnoid hemorrhage. Stroke 38: 2858–2860. 17761923 10.1161/STROKEAHA.107.485441

[41] Cramer SC (2008) Repairing the human brain after stroke: I. Mechanisms of spontaneous recovery. Ann Neurol 63: 272–287. 18383072 10.1002/ana.21393

[42] Cramer SC, Procaccio V, GAIN Americas, GAIN International Study Investigators (2012) Correlation between genetic polymorphisms and stroke recovery: Analysis of the GAIN Americas and GAIN International Studies. Eur J Neurol 19: 718–24. 22221491 10.1111/j.1468-1331.2011.03615.x

[43] Ventriglia M, Bocchio Chiavetto L, Binetti G, Zanetti O, et al (2002) Association between the BDNF 196 A/G polymorphism and sporadic Alzheimer ’ s. Mol Psychiatry 4: 8–9. 10.1038/sj.mp.4000952 11840305

[44] Sen S, Nesse RM, Stoltenberg SF, Li S, Gleiberman L, et al (2003) A BDNF coding variant is associated with the NEO personality inventory domain neuroticism, a risk factor for depression. Neuropsychopharmacology 28: 397–401. 12589394 10.1038/sj.npp.1300053

[45] Ribasés M, Gratacòs M, Armengol L, de Cid R, Badía A, et al (2003) Met66 in the brain-derived neurotrophic factor (BDNF) precursor is associated with anorexia nervosa restrictive type. Mol Psychiatry 8: 745–751. 12888803 10.1038/sj.mp.4001281

[46] Neves-Pereira M, Mundo E, Muglia P, King N, Macciardi F, et al (2002) The brain-derived neurotrophic factor gene confers susceptibility to bipolar disorder: evidence from a family-based association study. Am J Human Genet 71: 651–655. 12161822 10.1086/342288 PMC379201

